# The Influence of Performance Status, Inflammation, and Nutrition on the Impact of SGLT2 Inhibitors on Cancer Outcomes

**DOI:** 10.1002/cam4.70807

**Published:** 2025-03-21

**Authors:** Diego Rivas‐Otero, Tomás González‐Vidal, Pablo Agüeria‐Cabal, Guillermo Ramos‐Ruiz, Paula Jimenez‐Fonseca, Carmen Lambert, Pedro Pujante Alarcón, Edelmiro Menéndez Torre, Miguel García‐Villarino, Elías Delgado Álvarez

**Affiliations:** ^1^ Department of Endocrinology and Nutrition Central University Hospital of Asturias/University of Oviedo Oviedo Spain; ^2^ Endocrinology, Nutrition, Diabetes and Obesity (ENDO) Group Health Research Institute of Principality of Asturias/Instituto de Investigación Sanitaria del Principado de Asturias (ISPA) Oviedo Spain; ^3^ Department of Medicine University of Oviedo Oviedo Spain; ^4^ Department of Medical Oncology Central University Hospital of Asturias Oviedo Spain; ^5^ Rare Disease and Neoplasm Group Health Research Institute of Principality of Asturias/Instituto de Investigación Sanitaria del Principado de Asturias (ISPA) Oviedo Spain; ^6^ Centre for Biomedical Network Research on Rare Diseases (CIBERER) Carlos III Health Institute Madrid Spain

**Keywords:** cancer, mortality, progression, SGLT2, SGLT2 inhibitors

## Abstract

**Background:**

Sodium–glucose‐linked transporter 2 inhibitors (SGLT2i) may have antitumor effects. Previous studies analyzing their role in mortality and progression did not account for potential confounders, including cancer treatment, performance status, inflammatory markers, and nutritional status. This study aims to evaluate the impact of SGLT2i treatment on mortality and progression while considering these potential confounders.

**Methods:**

A retrospective cohort study was conducted. A total of 526 patients with cancer (302 women, mean age 64 years, range 40–79 years) were divided into two cohorts based on whether they were taking SGLT2i at the time of their cancer diagnosis and followed for 1 year or until death. All patients on SGLT2i were taking these drugs at standard clinical doses. Additional data collected included basic demographic variables, metabolic and lifestyle characteristics, cancer treatment received, performance status, inflammatory markers, and nutritional status. The primary endpoints were mortality and progression.

**Results:**

Patients taking SGLT2i at the time of cancer diagnosis (*n* = 41) were more likely to have type 2 diabetes, to be male, to be ever‐smokers, and to be older than patients not taking SGLT2i. In univariate analyses, SGLT2i treatment at the time of cancer diagnosis was not associated with mortality or cancer progression. Instead, mortality and cancer progression were positively associated with a diagnosis of T2D, male sex, older age, heavy alcohol drinking, ever‐smoker status, poor performance status, increased inflammation, malnutrition, tumor site, cancer stage, and lack of cancer treatment. After adjusting for these potential confounders, SGLT2i treatment was not significantly associated with mortality or cancer progression.

**Conclusions:**

Our results suggest that the impact of SGLT2i treatment on cancer outcomes is limited under standard clinical dosing conditions.

## Graphical abstract

This study highlights the critical role of patients‘ baseline characteristics, including performance status, inflammatory markers and nutritional status, in determining cancer mortality or progression. Our results showed both a nonsignificant effect of SGLT2 inhibitors on cancer mortality or progression and a nonsignificant effect of SGLT2 inhibitors on performance status, inflammatory markers, and malnutrition. These findings suggest that the potential benefits of SGLT2 inhibitors in cancer treatment may be constrained under standard clinical dosing conditions.

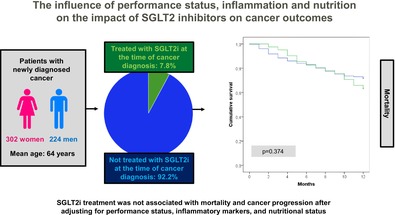

## 1 Introduction

The potential antitumor properties of antidiabetic drugs are an area of growing interest. Metformin was the first antidiabetic agent to show promising antitumor effects across various cancer types, although these effects may be limited by the need for high‐dose concentrations [1]. This paved the way for the investigation of other antidiabetic agents, such as sodium–glucose‐linked transporter 2 (SGLT2) inhibitors (SGLT2i), which are a family of drugs that increase the urinary excretion of glucose and sodium [2]. While SGLT2i were originally developed to treat diabetes, they have also been proven effective in the treatment of heart failure [3, 4, 5, 6, 7] and chronic kidney disease [8, 9, 10]. Several mechanisms have been proposed to explain the cardiorenal benefits of SGLT2i in patients with or without diabetes [11], including an indirect epigenetic effect [12]. This epigenetic mechanism may result from increased ketogenesis associated with SGLT2i [13], as elevated levels of β‐hydroxybutyrate can lead to histone modifications that help prevent inflammation and glucotoxicity [12]. More recently, SGLT2i have been suggested to exert potential antitumor effects [14, 15, 16]. However, the role of SGLT2i in the treatment of cancer in real‐life conditions remains unknown.

Cancer cells rely heavily on glucose as their main source of energy [17]. Laboratory‐based studies showed that cancer cells from diverse tissue types express SGLT2 receptors, as well as sodium–glucose‐linked transporter 1 receptors [14]. These findings prompted real‐life research to evaluate whether SGLT2i could play a role in cancer prevention or treatment. One set of studies focused on determining whether there is an association between SGLT2i treatment and the incidence of new cancer diagnoses. Although most of these studies found no significant association between SGLT2i treatment and cancer incidence [14, 18, 19, 20, 21, 22, 23, 24, 25], some suggested a reduced risk of organ‐specific [26] and overall [27, 28] cancer incidence in patients treated with these drugs. Despite these promising results, previous research also raised concerns about an increased risk of kidney cancer in patients taking SGLT2i [25]. Another set of studies investigated the potential role of SGLT2i as a component of an antitumor treatment regimen [28, 29, 30, 31, 32]. These studies showed reduced mortality rates in patients with cancer taking SGLT2i [28, 29], particularly in those with non–small cell lung cancer [30], liver cancer [31], and colorectal cancer [32]. However, these studies did not take into account other important confounding factors that could affect the results.

The cancer treatment received is a critical factor in cancer survival and progression rates, and not all previous studies adjusted for this covariate [28, 30, 31]. In two previous studies that accounted for cancer treatment received, patients taking iSGLT2 were more likely to undergo surgery, which may explain the better outcomes observed [30, 31]. Similarly, several previous studies did not adjust for performance status [28, 30, 31], another important determinant of cancer outcomes, because the cancer treatment chosen is highly dependent on performance status [33, 34] and because poor performance status is itself associated with decreased survival [35, 36]. In addition, it is known that physicians are less inclined to prescribe SGLT2i to patients at high risk of frailty [37], which could further skew the results. Indeed, patients treated with SGLT2i were younger in some of the studies showing beneficial effects of these drugs on cancer outcomes [30, 31]. Similarly, the majority of the aforementioned studies [29, 30, 31, 32] did not account for patients' inflammatory status. New tools have emerged to assess inflammatory status, such as the systemic inflammation response index (SIRI). An elevated SIRI indicates an increase in neutrophils and/or monocytes and a decrease in lymphocytes, reflecting an imbalance between pro‐ and anti‐inflammatory factors [38, 39, 40]. Similar to classic inflammatory markers such as hypoalbuminemia, an elevated SIRI has prognostic value for poor cancer outcomes [38, 39, 40, 41]. Hypoalbuminemia is not only a marker of inflammation, but may also indicate poor nutritional status [41, 42, 43]. Malnutrition and weight loss are other well‐known predictors of mortality in patients with cancer [44, 45, 46, 47] that were also not taken into account in some previous studies evaluating the impact of SGLT2i on cancer outcomes [28, 29, 30, 31, 32], even considering that SGLT2i are known to favor weight loss [3, 5, 7].

It is therefore unclear whether SGLT2i are useful in the treatment of cancer. The present study aims to be the first to evaluate the impact of SGLT2i treatment on cancer mortality and progression while adjusting for patients' performance status, inflammatory markers, and nutritional status. By addressing these potential confounding factors, we aim to provide a more accurate assessment of the relationship between SGLT2i use and cancer outcomes.

## 2 Patients and Methods

### 2.1 Study Design and Population

**FIGURE 1:**
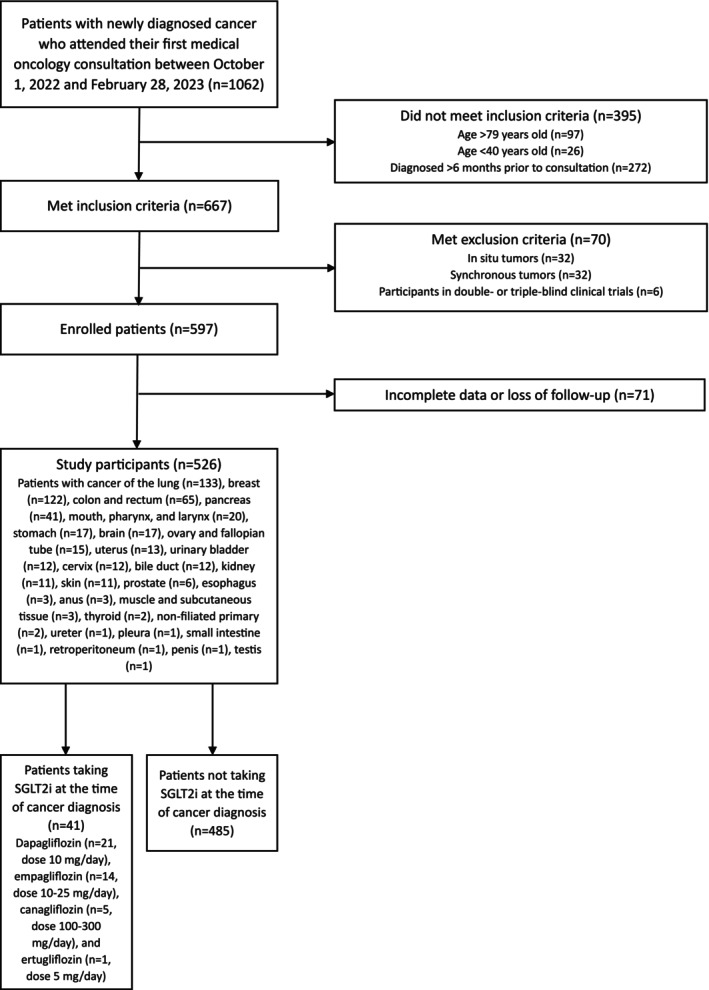
Flowchart of the study. SGLT2i, sodium–glucose‐linked transporter 2 inhibitors.

A retrospective cohort study was conducted at the Central University Hospital of Asturias, a tertiary care center in Spain. All patients who attended their first medical oncology consultation between October 1, 2022, and February 28, 2023, were consecutively preselected. The eligible population included individuals aged 40–79 years with a newly diagnosed cancer within the previous 6 months, and only those with data for a 1‐year follow‐up period or until the occurrence of death were included. The excluded population consisted of patients with in situ tumors, patients with synchronous tumors, participants in double‐ or triple‐blind clinical trials, and patients with hematological malignancies, to ensure cohort homogeneity. After applying these criteria, a total of 526 patients (224 men, mean age 64 years [standard deviation 9 years]) were ultimately enrolled and followed for 1 year (Figure 1). The sample was divided into two cohorts: one consisting of patients who were taking SGLT2i at the time of cancer diagnosis (*n* = 41), and the other consisting of patients who were not taking SGLT2i at the time of cancer diagnosis (*n* = 485). Figure 1 shows the cancer types of the study participants and the daily doses of SGLT2i they were taking. No patient was taking doses of SGLT2i that exceeded the maximum approved daily doses (Figure 1) [48].

### 2.2 Data Collection

Patient characteristics collected at the time of cancer diagnosis included age, sex, body mass index (BMI), performance status using the Eastern Cooperative Oncology Group Performance Status (ECOG‐PS) score [35], smoking history (current and former smokers were considered ever‐smokers), and alcohol consumption (patients who reported drinking > 30 g/day of alcohol were considered heavy alcohol drinkers). Metabolic characteristics recorded included known diagnosis of type 2 diabetes (T2D) prior to cancer diagnosis and treatment with different antidiabetic agents at the time of cancer diagnosis: SGLT2i, metformin, dipeptidyl peptidase‐4 inhibitors (DPP4i), glucagon‐like peptide‐1 receptor agonists (GLP‐1 RA), and insulin. Laboratory data collected at the time of cancer diagnosis included neutrophil, lymphocyte, and monocyte counts to calculate the SIRI (neutrophils x monocytes/lymphocytes) [38], and serum albumin concentrations. Cancer‐related variables included histological type, primary cancer site, and stage at diagnosis [49]. Cancers of the pancreas, lung, pleura, brain, esophagus, stomach, and bile duct were considered high‐mortality cancers in some analyses [50]. The following data were collected during the 1‐year follow‐up period: cancer treatment received (cancer surgery, chemotherapy, radiotherapy, and/or immunotherapy), change in BMI at 3–6 months (preferably at 3 months) compared with BMI at the time of cancer diagnosis (%BMI, calculated as [[BMI at 3–6 months/BMI at diagnosis] x 100]), cancer progression, and all‐cause mortality. Cancer progression and mortality were the primary endpoints of the study. Cancer progression was defined by radiologic criteria using the Response Evaluation Criteria in Solid Tumors (RECIST) [51]. Survival was calculated in months from diagnosis to death from any cause, and progression‐free survival was calculated in months from diagnosis to progression or death, whichever occurred first.

### 2.3 Statistical Analyses

Categorical variables were compared using the chi‐square test (X^2^) and continuous variables were compared using Student's *t*‐test. Kaplan–Meier survival analysis, along with the log‐rank test, was used to evaluate differences in survival and progression‐free survival according to the use of SGLT2i at the time of cancer diagnosis. Additionally, Cox proportional hazards regression models were used for multivariate analysis of survival and progression‐free survival, adjusting for potential confounders. All statistical tests were two‐tailed, and a *p* value of less than 0.05 was considered indicative of statistical significance. The analyses were performed using IBM SPSS Statistics, version 21.

## 3 Results

Of the 526 patients recruited, 41 (7.7%) were taking SGLT2i at the time of their cancer diagnosis. As expected, patients taking SGLT2i at the time of cancer diagnosis had a higher frequency of T2D than those not taking SGLT2i (Table 1). In fact, 92.7% (38/41) of the patients who were taking SGLT2i at the time of cancer diagnosis had a known diagnosis of T2D. The remaining three patients were taking SGLT2i for heart failure with reduced ejection fraction. Patients taking SGLT2i at the time of cancer diagnosis were also more likely to be male, to be ever‐smokers, and to be older than those not taking SGLT2i (Table 1).

**TABLE 1:** Characteristics of the recruited population.

Characteristic		SGLT2i(41)	No SGLT2i (485)	*p*	Death (152)	No death (374)	*p*	Progress (218)	No progress (308)	*p*
Sex (man)		26 (63.4)	198 (40.8)	**0.008**	90 (59.2)	134 (35.8)	**< 0.001**	127 (58.3)	97 (31.5)	**< 0.001**
Age at diagnosis (years)		67 ± 7	64 ± 10	**0.043**	67 ± 7	63 ± 10	**< 0.001**	66 ± 8	62 ± 10	**< 0.001**
Type 2 diabetes (yes)		38 (92.7)	74 (15.3)	**< 0.001**	47 (30.9)	65 (17.4)	**0.001**	62 (28.8)	50 (16.2)	**0.001**
SGLT2i at diagnosis (yes)		NA	NA		15 (9.9)	26 (7.0)	0.283	21 (9.6)	20 (6.5)	0.191
ECOG‐PS ≥ 2 (yes)^a^		7 (17.1)	71 (14.6)	0.821	58 (38.2)	20 (5.3)	**< 0.001**	64 (29.4)	14 (4.5)	**< 0.001**
Ever‐smoker (yes)^b^		33 (80.4)	309 (63.7)	**0.040**	122 (80.2)	220 (58.8)	**< 0.001**	171 (78.4)	171 (55.5)	**< 0.001**
Heavy alcohol drinker (yes)^b^		4 (9.7)	34 (7.0)	0.526	21 (13.8)	17 (4.5)	**0.001**	25 (11.4)	13 (4.2)	**0.002**
BMI at diagnosis (kg/m^2^)^c^		28.0 ± 6.0	26.7 ± 5.2	0.149	26.3 ± 4.8	27.0 ± 5.5	0.161	26.2 ± 5.1	27.3 ± 5.4	**0.026**
BMI categories at diagnosis^c^	Low normal (< 25.0 kg/m^2^)	12 (29.2)	196 (40.4)	0.154	67 (44.0)	141 (37.7)	0.278	102 (46.7)	106 (34.4)	**0.008**
	Overweight (25– < 30 kg/m^2^)	20 (48.7)	163 (33.6)		46 (30.2)	137 (36.6)		64 (29.3)	119 (38.6)	
	Obesity ( ≥ 30 kg/m^2^)	8 (19.5)	107 (22.0)		31 (20.3)	84 (22.4)		41 (18.8)	74 (24.0)	
%BMI^d^		99.04 ± 8.07	98.63 ± 7.38	0.760	96.35 ± 7.15	99.24 ± 7.39	**0.001**	97.57 ± 7.08	99.24 ± 7.56	**0.026**
Albumin at diagnosis (g/L)^e^		40 ± 6	40 ± 5	0.997	38 ± 5	41 ± 5	**< 0.001**	38 ± 5	42 ± 5	**< 0.001**
SIRI at diagnosis^f^		3.8 ± 4.1	4.1 ± 17.1	0.910	7.1 ± 27.6	2.8 ± 7.6	**0.008**	6.6 ± 24.8	2.2 ± 3.2	**0.003**
Advanced stage at diagnosis (yes)^g^		31 (75.6)	323 (66.6)	0.299	147 (96.7)	207 (55.3)	**< 0.001**	206 (94.5)	148 (48.1)	**< 0.001**
Primary tumor site	Colorectal	7 (17.1)	58 (12.0)	0.059	8 (5.3)	57 (15.2)	**< 0.001**	20 (9.2)	45 (14.6)	**< 0.001**
	Lung	13 (31.7)	120 (24.7)		70 (46.1)	63 (16.8)		97 (44.5)	36 (11.7)	
	Breast	3 (7.3)	119 (24.5)		0 (0.0)	122 (32.6)		1 (0.5)	121 (39.3)	
	Pancreas	6 (14.6)	35 (7.2)		27 (17.8)	14 (3.7)		27 (12.4)	14 (4.5)	
	Other	12 (29.3)	153 (31.5)		47 (30.9)	118 (31.6)		73 (33.5)	92 (29.9)	
High‐mortality cancer (yes)^h^		22 (53.7)	202 (41.6)	0.142	114 (75.0)	110 (29.4)	**< 0.001**	149 (68.3)	75 (24.4)	**< 0.001**
Cancer histological type	Adenocarcinoma	24 (58.5)	308 (63.5)	0.826	79 (51.9)	253 (67.6)	**< 0.001**	109 (50.0)	223 (72.4)	**< 0.001**
	Squamous	6 (14.6)	63 (12.9)		24 (15.7)	45 (12.0)		35 (16.0)	34 (11.0)	
	Neuroendocrine	4 (9.7)	31 (6.3)		20 (13.1)	15 (4.0)		28 (12.8)	7 (2.2)	
	Other	6 (14.6)	76 (15.6)		25 (16.4)	57 (15.2)		39 (17.8)	43 (13.9)	
Cancer surgery (yes)		21 (51.2)	262 (54.0)	0.747	17 (11.2)	266 (71.1)	**< 0.001**	40 (18.3)	243 (78.9)	**< 0.001**
Chemotherapy (yes)		27 (65.9)	292 (60.2)	0.510	77 (50.7)	242 (64.7)	**0.003**	129 (59.2)	190 (61.7)	0.587
Radiotherapy (yes)		15 (36.6)	249 (51.3)	0.075	58 (38.2)	206 (55.1)	**0.001**	97 (44.5)	167 (54.2)	**0.034**
Immunotherapy (yes)		10 (24.4)	134 (27.6)	0.719	36 (23.7)	108 (28.9)	0.237	75 (34.4)	69 (22.4)	**0.003**

*Note:* Data are expressed as absolute numbers and percentage (within parentheses) or as mean ± standard deviation. *p* < 0.05 are highlighted in bold.

Abbreviations: BMI, body mass inde; ECOG‐PS, Eastern Cooperative Oncology Group Performance Status; NA, not applicable; SGLT2i, sodium–glucose‐linked transporter 2 inhibitors; SIRI, systemic inflammation response index.

^a^ Data are available for 490 patients.

^b^ Data available for 523 patients. Current and former smokers were considered ever‐smokers. Patients who reported drinking > 30 g/day of alcohol were considered heavy drinkers.

^c^ Data available for 506 patients.

^d^ Data available for 432 patients. %BMI was calculated as ([BMI at 3–6 months/BMI at diagnosis] x 100).

^e^ Data available for 349 patients.

^f^ Data available for 503 patients. The SIRI was calculated as (neutrophil count × monocyte count/lymphocyte count) from blood samples collected at the time of cancer diagnosis.

^g^ ^“^Yes” in patients who were diagnosed with Stage III or IV cancer.

^h^ Cancers of the pancreas, lung, pleura, brain, esophagus, stomach, and bile duct were considered to have a high mortality rate.

During the 1‐year follow‐up period, 152 (28.8%) patients died (first primary endpoint) and 218 (41.4%) experienced cancer progression or death (second primary endpoint). Patients who were taking SGLT2i at the time of cancer diagnosis had similar mortality and progression rates to those who were not taking SGLT2i (Table 1). Instead, mortality and cancer progression were associated with older age, male sex, diagnosis of T2D, worse performance status, ever‐smoker status, heavy alcohol drinking, lower BMI or %BMI, and higher inflammatory status (including a higher SIRI and lower serum albumin concentrations). As expected, primary tumor site, histologic cancer type, tumor stage at diagnosis, and cancer treatments received were also associated with mortality and cancer progression (Table 1). Given that male sex was associated with both poor cancer outcomes and the use of SGLT2i (Table 1), a sex‐stratified analysis was performed to evaluate the impact of SGLT2i on cancer mortality and progression (Table S1). Again, this analysis showed no significant association between SGLT2i use and cancer outcomes.

**FIGURE 2:**
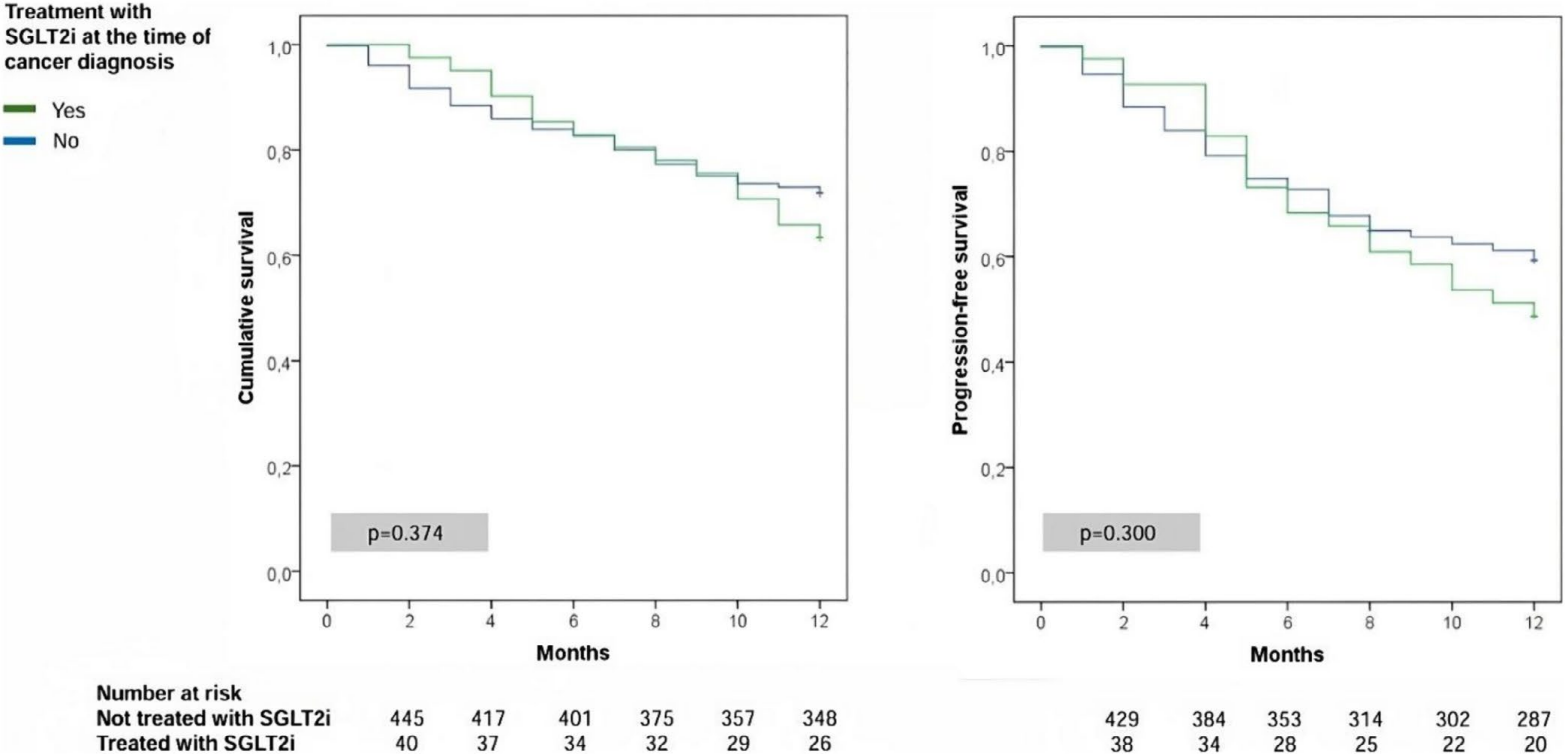
Kaplan–Meier analysis for mortality (left panel) and cancer progression (right panel).

Univariate survival analyses further showed that the use of SGLT2i at the time of cancer diagnosis was not associated with mortality or cancer progression (Figure 2). In multivariate survival analyses (Cox regression), the use of SGLT2i at the time of cancer diagnosis was also not significantly associated with mortality or cancer progression (Table 2). Similarly, the use of other antidiabetic agents was also not associated with these cancer outcomes. In contrast, receiving any type of cancer treatment (including cancer surgery, radiotherapy, chemotherapy, or immunotherapy) remained associated with better cancer outcomes (Table 2). Low‐serum albumin levels, impaired performance status (ECOG‐PS ≥ 2), cancers with known high mortality rates [50], and advanced stage at diagnosis (Stage III or IV) also maintained a positive association with mortality and cancer progression in all regression models (Table 2).

**TABLE 2:** Multivariate analyses of factors associated with mortality and cancer progression (Cox regression).

		Death			Progression		
		Hazard ratio	95% CI	*p*	Hazard ratio	95% CI	*p*
Model 1	Sex (man)	1.43	1.01–2.04	**0.043**	1.58	1.17–2.12	**0.002**
	Age at diagnosis (years)	1.04	1.02–1.06	**< 0.001**	1.02	1.01–1.04	**0.001**
	Type 2 diabetes (yes)	1.57	1.06–2.33	**0.023**	1.40	0.99–1.97	0.053
	SGLT2i at diagnosis (yes)	0.65	0.35–1.20	0.174	0.71	0.42–1.19	0.203
	Ever‐smoker (yes)^a^	2.20	1.42–3.39	**< 0.001**	1.94	1.37–2.75	**< 0.001**
Model 2	Sex (man)	1.01	0.66–1.55	0.940	1.03	0.72–1.47	0.850
	Age at diagnosis (years)	1.01	0.98–1.03	0.389	1.00	0.98–1.02	0.617
	Type 2 diabetes (yes)	1.24	0.74–2.08	0.399	1.05	0.67–1.63	0.815
	BMI at diagnosis (kg/m^2^)	1.01	0.98–1.05	0.384	1.00	0.97–1.03	0.926
	Serum albumin at diagnosis (g/L)	0.95	0.92–0.99	**0.015**	0.96	0.94–0.99	**0.025**
	SIRI at diagnosis (score)^b^	1.00	0.99–1.00	0.657	1.00	0.99–1.00	0.351
	ECOG‐PS ≥ 2 (yes)	2.33	1.36–3.97	**0.002**	1.70	1.06–2.75	**0.028**
	High‐mortality cancer (yes)^c^	2.38	1.50–3.79	**< 0.001**	1.86	1.30–2.68	**0.001**
	Advanced stage at diagnosis (yes)^d^	6.26	1.95–20.13	**0.002**	6.54	2.84–15.06	**< 0.001**
	Any cancer treatment (yes)^e^	0.14	0.07–0.28	**< 0.001**	0.23	0.12–0.42	**< 0.001**
	SGLT2i at diagnosis (yes)	0.74	0.36–1.51	0.416	0.87	0.48–1.57	0.654
	Ever‐smoker (yes)^a^	1.49	0.89–2.49	0.123	1.43	0.95–2.16	0.083
	Heavy alcohol drinker (yes)^f^	1.84	1.00–3.38	**0.047**	1.40	0.82–2.39	0.213
Model 3	Sex (man)	1.10	0.76–1.58	0.601	1.18	0.88–1.59	0.261
	Age at diagnosis (years)	1.00	0.98–1.03	0.390	1.00	0.98–1.02	0.587
	ECOG‐PS ≥ 2 (yes)	2.42	1.48–3.93	**< 0.001**	1.75	1.15–2.67	**0.009**
	High‐mortality cancer (yes)^c^	2.57	1.73–3.84	**< 0.001**	2.08	1.53–2.84	**< 0.001**
	Advanced stage at diagnosis (yes)^d^	9.18	3.32–25.40	**< 0.001**	6.71	3.57–12.61	**< 0.001**
	Any cancer treatment (yes)^e^	0.12	0.07–0.23	**< 0.001**	0.20	0.12–0.34	**< 0.001**
	SGLT2i at diagnosis (yes)	0.70	0.33–1.48	0.358	0.78	0.41–1.46	0.445
	Metformin at diagnosis (yes)	1.29	0.73–2.29	0.369	1.03	0.63–1.68	0.892
	DPP4i at diagnosis (yes)	0.67	0.33–1.33	0.255	0.95	0.53–1.67	0.864
	GLP‐1 RA at diagnosis (yes)	0.62	0.20–1.84	0.390	1.13	0.46–2.78	0.782
	Insulin at diagnosis (yes)	1.35	0.65–2.80	0.408	0.94	0.48–1.84	0.877
	Heavy alcohol drinker (yes)^f^	1.88	1.12–3.15	**0.017**	1.54	0.97–2.43	0.063

*Note:* Model 1 included 523 patients. Model 2 included 321 patients. Model 3 included 489 patients. Serum albumin levels at diagnosis were not included in Model 3 because these data were available for only 349 patients. When serum albumin levels were included in Model 3, they maintained a negative association with cancer outcomes, while the rest of the model results did not change significantly (data not shown). *p* < 0.05 are highlighted in bold.

Abbreviations: BMI, body mass index; CI, confidence interval; DPP4i, dipeptidyl peptidase 4 inhibitors; ECOG‐PS, Eastern Cooperative Oncology Group Performance Status; GLP‐1 RA, glucagon‐like peptide‐1 agonists; SGLT2i, sodium–glucose‐linked transporter 2 inhibitors; SIRI, systemic inflammation response index.

^a^ Current and former smokers were considered ever‐smokers.

^b^ The SIRI was calculated as (neutrophil count × monocyte count/lymphocyte count) from blood samples collected at the time of cancer diagnosis.

^c^ Cancers of the pancreas, lung, pleura, brain, esophagus, stomach, and bile duct were considered to have a high mortality rate.

^d^ “Yes” in patients who were diagnosed with stage III or IV cancer.

^e^ “Yes” in patients who received any cancer treatment, including cancer surgery, radiotherapy, chemotherapy, or immunotherapy.

^f^ Patients who reported drinking > 30 g/day of alcohol were considered heavy drinkers.

## 4 Discussion

The objective of this study was to investigate the impact of SGLT2i treatment on cancer outcomes (mortality and progression). To our knowledge, this study is the first to evaluate the impact of SGLT2i treatment on mortality and cancer progression while controlling for potential confounding factors, including performance status, inflammatory markers, and nutritional status. Despite the initial hypothesis suggesting that treatment with SGLT2i might influence cancer outcomes, our results showed no significant difference in cancer survival or progression between patients who received SGLT2i and those who did not.

Univariate analyses in our study showed that patients taking SGLT2i at the time of cancer diagnosis did not have a higher frequency of mortality or cancer progression during 1 year of follow‐up. However, SGLT2i users had a higher frequency of T2D and were more likely to be male, ever‐smokers, and older than those not taking SGLT2i. All these factors associated with SGLT2i treatment were also associated with poor cancer outcomes. However, after adjusting for these potential confounders (Table 2, Model 1), we found that SGLT2i treatment was not significantly associated with cancer mortality and progression.

Standardized scales such as ECOG‐PS and the Karnofsky Scale are often used by oncologists to assess the overall condition of patients [35, 36]. These scales are useful in guiding cancer treatment as they provide information on the tolerability of such treatments, especially in patients older than 65 years [34]. In our study, and consistent with previous studies showing that ECOG‐PS has prognostic value for mortality [35], impaired performance status at diagnosis (defined as ECOG‐PS ≥ 2) was independently associated with poor cancer outcomes. For these reasons, performance status is a confounding factor that needs to be controlled for when evaluating the impact of drugs such as SGLT2i on cancer outcomes. Some previous studies reporting beneficial effects of SGLT2i on cancer survival did not adjust for this important covariate [28, 30, 31]. In our study, SGLT2i use at cancer diagnosis was also not associated with mortality or cancer progression after adjusting for performance status. Contrary to our results, some previous studies showed beneficial effects of SGLT2i in cancer after adjusting for performance status [29, 32]. However, these [29, 32] and other studies [28, 30, 31] did not account for other variables that were also associated with mortality in our study, such as inflammatory status [29, 30, 31, 32] and nutritional status [28, 29, 30, 31, 32].

Classic and novel inflammatory markers, such as hypoalbuminemia and an elevated SIRI, respectively, are known to be associated with increased mortality in patients with cancer [38, 39, 52, 53, 54, 55, 56]. In our study, both low‐serum albumin concentrations and an elevated SIRI were associated with mortality and cancer progression in univariate analyses (Table 1), but only albumin remained associated with these poor cancer outcomes in multivariate analyses (Table 2). In contrast to SIRI, hypoalbuminemia might also be considered a classic marker of nutritional status [41, 42, 43], although its usefulness in assessing malnutrition is currently much debated [57, 58]. This possible dual meaning of serum albumin levels may give hypoalbuminemia a superior prognostic value for cancer outcomes, as nutritional status is an established prognostic factor in cancer. The prevalence of malnutrition in patients with cancer is as high as 76% [59]. Previous studies showed higher mortality rates in patients with cancer who lose significant weight after surgery [47], during chemotherapy [44, 45], or during radiotherapy [46]. In our study, weight loss during follow‐up (reported as %BMI) was also higher in patients who died or experienced cancer progression. Similarly, patients with low‐normal BMI (< 25 kg/m^2^) at the time of cancer diagnosis had high mortality and progression rates. Although obesity (BMI ≥ 30 kg/m^2^) is another known risk factor for cancer progression in certain cancers [60], our results showed that the frequency of progression was higher in patients with low‐normal BMI than in those with obesity. This finding may be explained by unmeasured factors such as cancer‐related cachexia, which is particularly common in patients with advanced disease [61]. Larger studies are needed to compare the impact of low‐normal BMI versus obesity in each cancer type. Although SGLT2i have the potential to exert anti‐inflammatory effects [12] and promote moderate weight loss [3, 5, 7, 62], our results showed that both inflammatory status at diagnosis and changes in BMI during follow‐up were similar in SGLT2i users and nonusers. Furthermore, after adjusting for these potential confounders, SGLT2i use at the time of cancer diagnosis was not significantly associated with mortality or cancer progression.

A major limitation of our study is the observational design, which limits our ability to establish a causal relationship between SGLT2i use and cancer outcomes. The study encompasses a diverse range of tumor types and has a relatively small sample size for each one, which hindered the analysis of the effect of SGLT2i treatment in each individual cancer type. In particular, it would have been of interest to assess the role of SGLT2i on kidney cancer survival and progression, as there is growing concern about an increased incidence of kidney cancer in patients treated with these drugs [25, 63]. The impact of SGLT2i on cancer outcomes was assessed by whether patients were taking the drug at the time of cancer diagnosis, without analyzing whether they continued to take SGLT2i for the remainder of the follow‐up period. We chose this intention‐to‐treat approach to avoid confounding by indication bias. As shown in Table S2, the few patients who discontinued or started SGLT2i during follow‐up had unequal frequencies of poor cancer outcomes (particularly, mortality). Patients who were taking SGLT2i at the time of cancer diagnosis, but who discontinued these drugs during follow‐up (*n* = 8), had a high frequency of poor cancer outcomes (possibly they were de‐intensified from antidiabetic treatment due to their poor clinical condition, which may have made antidiabetic treatment a low priority). Consistent with previous studies showing that SGLT2i are less likely to be started in fragile individuals [37], patients not taking SGLT2i at cancer diagnosis who started these drugs during follow‐up (*n* = 12) had a low frequency of poor cancer outcomes (possibly they were intensified on antidiabetic treatment because their clinical condition was good, allowing prioritization of diabetes control). Future prospective studies with longitudinal data on the use of antidiabetic drugs after diagnosis are needed to fill this gap. Additionally, we did not evaluate the effect of the duration of exposure to SGLT2i prior to the cancer diagnosis, the impact of concurrent use of other common cancer drugs like corticosteroids, or whether there is a synergistic effect with antitumor agents. This highlights the need for larger studies to validate our findings and explore additional variables that could influence outcomes in patients with cancer taking SGLT2i. Although our study did not find a significant association between cancer outcomes and the use of SGLT2i at doses commonly used in clinical practice [48], both previous laboratory‐based and real‐life studies suggested that the beneficial effects of SGLT2i for cancer treatment may be dose‐dependent [14, 29, 30, 31]. Further research is needed to determine the optimal dose of SGLT2i to achieve a potential antitumor effect.

In conclusion, our study highlights the critical impact of performance status, inflammatory markers, and nutritional status on cancer outcomes. Our results showed a nonsignificant association between SGLT2i and performance status, inflammatory markers, and malnutrition. After adjusting for these confounders, SGLT2i were not associated with either mortality or cancer progression during 1 year of follow‐up. These findings suggest that the potential benefits of SGLT2i in cancer treatment may be limited under standard clinical dosing conditions. While the potential antitumor effects of SGLT2i remain a topic of interest, larger prospective studies are needed to validate these results after adjusting for other potential confounders and to evaluate the effect of higher doses of SGLT2i on cancer outcomes.

## Author Contributions

**Diego Rivas‐Otero:** conceptualization, investigation, writing – original draft, methodology, visualization, writing – review and editing, software, formal analysis, project administration, data curation, resources. **Tomás González‐Vidal:** methodology, validation, visualization, writing – review and editing, software, formal analysis, data curation, supervision, resources. **Pablo Agüeria‐Cabal:** data curation. **Guillermo Ramos‐Ruiz:** data curation. **Paula Jimenez‐Fonseca:** investigation, methodology, validation, writing – review and editing, supervision, resources. **Carmen Lambert:** supervision. **Pedro Pujante Alarcón:** supervision. **Edelmiro Menéndez Torre:** supervision. **Miguel García‐Villarino:** validation, writing – review and editing, formal analysis, supervision, resources. **Elías Delgado Álvarez:** supervision.

## Ethics Statement

This study was conducted in accordance with the Declaration of Helsinki and the Committee on Publication Ethics (COPE) and was approved by the regional ethics committee of the Principado de Asturias (CEImPA), code CEImPA 2024.070. According to national legislation and institutional guidelines, written informed consent of study participants was not required. All data were anonymized to ensure participant confidentiality and any personal identifiers were removed to maintain privacy.

## Conflicts of Interest

Diego Rivas‐Otero reports financial support for scientific meetings from Novo Nordisk, Lilly, Sanofi, Menarini, and Ypsomed. Tomás González‐Vidal reports financial support for scientific meetings from Novo Nordisk, Lilly, and Sanofi. Pablo Agüeria‐Cabal reports financial support for scientific meetings from Lilly and Sanofi. Guillermo Ramos‐Ruiz reports financial support for scientific meetings from Lilly and Insulcloud. Elías Delgado Álvarez reports unrestricted research support from AstraZeneca, Novo Nordisk, Sanofi, Pfizer, and Roche and has received consulting fees and/or honoraria for membership on advisory boards and speaker's bureau from AstraZeneca, Novo Nordisk, Lilly, Sanofi, GlaxoSmithKline, Pfizer, Almirall, Novartis, Abbott Laboratories, Esteve, and Merck Sharp & Dohme. Pedro Pujante Alarcón reports speaker's bureau from Novo Nordisk, Lilly, Sanofi, and Abbott Laboratories. The remaining authors have no conflicts of interest related to the scope of this study, the study design, or its results.

## Supporting information


Tables S1–S2.

## Data Availability

The data that support the findings of this study are available from the corresponding author upon reasonable request.
