# Supplementary material for: The Influence of Performance Status, Inflammation, and Nutrition on the Impact of SGLT2 Inhibitors on Cancer Outcomes
Source: Cancer Med. 2025 Mar 21;14(6):e70807. doi: 10.1002/cam4.70807 (PMC11926916; doi:10.1002/cam4.70807)
Supplement: Supplementary file 1 — Tables S1–S2. [file CAM4-14-e70807-s001.docx]

**Supplementary Table 1.** Sex-stratified analysis of the association between SGLT2i treatment and cancer outcomes.

| **Female patients** (n=302) | | | |
| --- | --- | --- | --- |
| **Cancer outcome** | **Treatment with SGLT2i at diagnosis** (n=15) | **No treatment with SGLT2i at diagnosis** (n=287) | **P value** |
| **Death** (yes) | 5 (33.3) | 57 (19.9) | 0.202 |
| **Progression** (yes) | 7 (46.7) | 84 (29.3) | 0.159 |

| **Male patients** (n=224) | | | |
| --- | --- | --- | --- |
| **Cancer outcome** | **Treatment with SGLT2i at diagnosis** (n=26) | **No treatment with SGLT2i at diagnosis** (n=198) | **P value** |
| **Death** (yes) | 10 (38.5) | 80 (40.4) | 1.000 |
| **Progression** (yes) | 14 (53.8) | 113 (57.1) | 0.834 |

Data are expressed as absolute numbers and percentage (within parentheses).

**Supplementary Table 2.** Cancer outcomes according to the use of SGLT2i during the entire follow-up period.

| **Cancer outcome** | **No treatment with SGLT2i at any time**  (n=473) | **Treatment with SGLT2i from baseline and throughout the entire follow-up period** (n=33) | **Baseline treatment with SGLT2i, but discontinued during follow-up**  (n=8) | **No baseline treatment with SGLT2i, but started during follow-up** (n=12) | **P value** |
| --- | --- | --- | --- | --- | --- |
| **Death** (yes) | 135 (28.5) | 10 (30.3) | 5 (62.5)* | 2 (16.7) | 0.149 |
| **Progression** (yes) | 191 (40.4) | 16 (48.5) | 5 (62.5) | 6 (50.0) | 0.437 |

Data are expressed as absolute numbers and percentage (within parentheses).
P-values were obtained with the Chi-squared test. *P <0.05 versus no treatment with SGLT2i at any time.
